# Toll-like receptors in breast cancer immunity and immunotherapy

**DOI:** 10.3389/fimmu.2024.1418025

**Published:** 2024-06-06

**Authors:** Joseph Zhou, Lin Zhang, Siyao Liu, David DeRubeis, Dekai Zhang

**Affiliations:** ^1^ Center for Infectious and Inflammatory Diseases, Institute of Biosciences and Technology, Texas A&M University, Houston, TX, United States; ^2^ Center for Translational Cancer Research, Institute of Biosciences and Technology, Texas A&M University, Houston, TX, United States

**Keywords:** Toll-like receptors, breast cancer, innate immunity, cancer immunity, immunotherapy, immune checkpoint inhibitor

## Abstract

Toll-like receptors (TLRs) are a key family of pattern recognition receptors (PRRs) in the innate immune system. The activation of TLRs will not only prevent pathogen infection but also respond to damage-induced danger signaling. Increasing evidence suggests that TLRs play a critical role in breast cancer development and treatment. However, the activation of TLRs is a double-edged sword that can induce either pro-tumor activity or anti-tumor effect. The underlying mechanisms of these opposite effects of TLR signaling in cancer are not fully understood. Targeting TLRs is a promising strategy for improving breast cancer treatment, either as monotherapies or by improving other current therapies. Here we provide an update on the role of TLRs in breast cancer immunity and immunotherapy.

## 1 Introduction

Toll-like receptors (TLRs) were originally discovered as a family of receptors to recognize pathogen-associated molecular patterns (PAMPs) from non-self (1). However, soon after their discovery, it was proposed that TLRs could also recognize damage-associated molecular patterns (DAMPs) from self (2). Furthermore, TLR signaling plays a critical role not only in activating innate immune responses but also in regulating adaptive immunity (3). Without TLR signaling, T-cell activation is feeble (3). Thus far, tremendous evidence has shown that TLRs and TLR signaling pathways play an important role in cancer development and treatment. Targeting TLRs holds great promise for developing a new therapeutic agent, or for improving the current treatments for breast cancer, or for developing a powerful anti-tumor vaccine as an adjuvant.

Breast cancer is the most common cancer for women worldwide, and the second leading cause of cancer-associated death after lung cancer. With the advancement of early diagnosis and better treatment options, the outcome of breast cancer patients has dramatically improved in recent years (4). However, for late-stage breast cancer patients, the efficacy of current treatments is limited; these treatments include cancer immunotherapy, such as immune checkpoint inhibitors targeting programmed cell death protein (PD-1) and cytotoxic T-lymphocyte-associated antigen 4 (CTLA-4) (5–7). Therefore, a novel strategy to develop a new therapeutic agent or improve the current treatment for breast cancer is urgently needed.

Increasing evidence has demonstrated that innate immunity, particularly TLRs, plays a pivotal role in the regulation of tumor microenvironment and controlling immune responses in tumor development and treatment. In this review article, first, the role of each human TLR in breast cancer will be summarized and analyzed, then the potential mechanisms and current understanding of different TLR activations that may induce an opposite effect on breast cancer development will be explored. Finally, we will summarize targeting TLRs to develop novel monotherapies or combinations of other therapies including immune checkpoint inhibitors.

## 2 Toll-like receptor snapshot

The human immune system is divided into two main categories: innate immunity and adaptive immunity. However, the importance of innate immunity had not been appreciated until the discovery of the first human Toll-like receptor (later assigned as human TLR4) in 1997 (8). In the following, the ligand LPS of this first-identified TLR was identified with nature mutant TLR4 mouse, that is bacterial lipopolysaccharides (LPS) (9). Then, people started realizing the importance of innate immunity, and the discovery of the Toll-like receptors was awarded the Nobel Prize in Medicine in 2011.

Toll-like receptors (TLRs) are a family of type I transmembrane proteins, and the first family of innate immunity receptors was defined as a family of pattern recognition receptors (PRRs). The basic structure of TLRs includes an extracellular leucine-rich repeat (LRR) domain, a transmembrane (TM) domain, and a cytoplasmic Toll/interleukin-1 receptor (TIR) domain. The LRR domain is responsible for ligand recognition; upon binding of ligands, TLR activation triggers the TLR signaling cascade. The TM domain allows TLRs to be embedded in the cell membrane. The TIR domains among TLRs are highly conserved, allowing them to activate the common signaling pathways: the MyD88-dependent pathway for all TLRs except TLR3, and the TRIF-dependent pathway for TLR3 and TLR4 (10). TLRs are evolutionarily conserved and mainly expressed on innate immune cells such as macrophages, dendritic cells, and neutrophils. TLRs are also expressed and activated on non-innate immune cells such as T cells, B cells, and epithelial cells (11). When TLRs recognize and bind to their ligands, they transfer the message into the cells to activate TLR signaling cascades. The ligands of TLRs are generally conserved and structurally essential molecular patterns in pathogens (called pathogen-associated molecular patterns, or PAMPs) or from damaged or dying cells (called damage-associated molecular patterns, or DAMPs) (12, 13).

**Figure 1:**
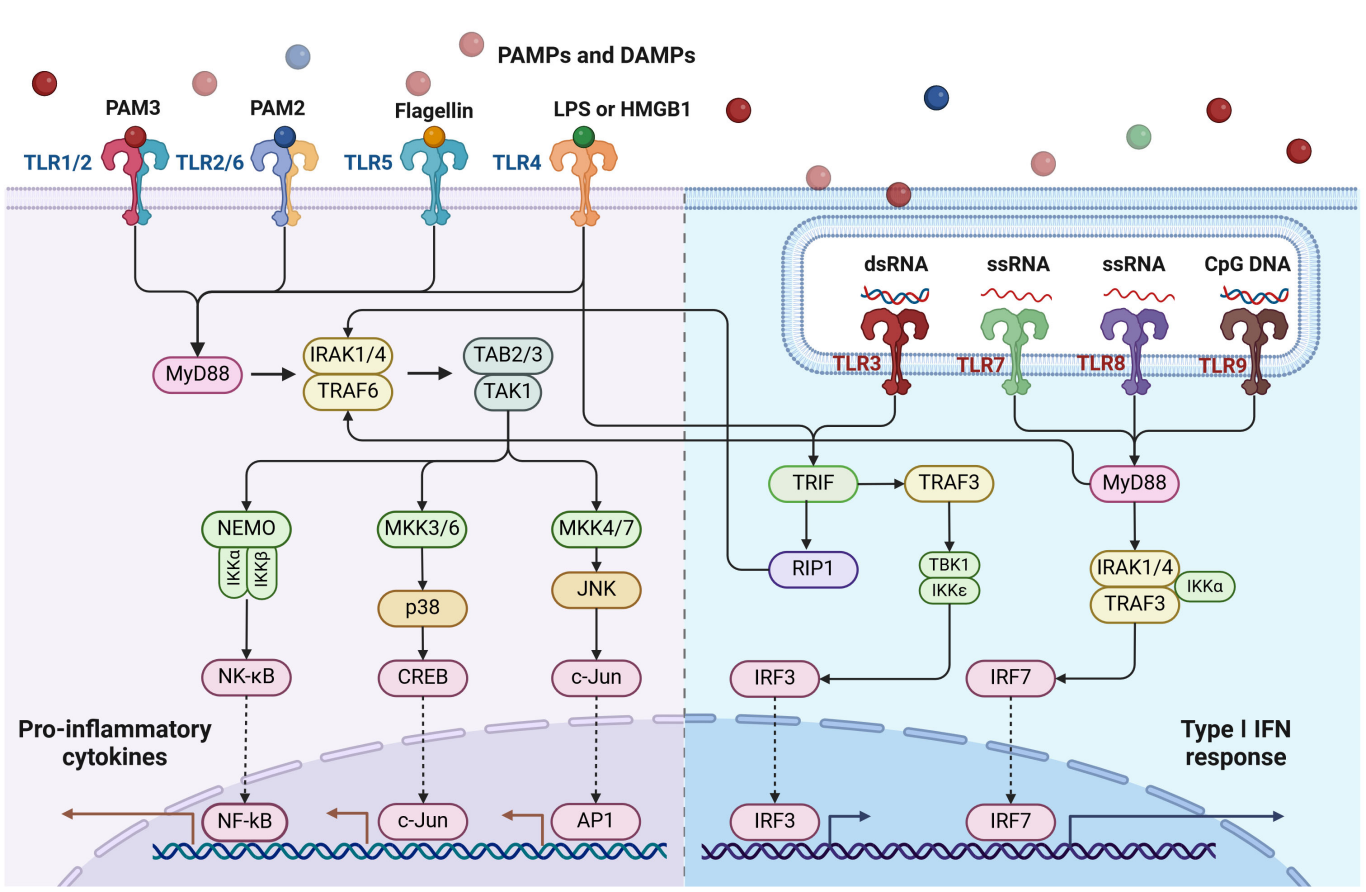
Toll-like receptors (TLRs) and their signaling pathways. TLRs are situated either on the cell surface (such as TLR1/2, TLR2/6, TLR4, TLR5) or intracellularly (like TLR3, TLR7/8, TLR9). With the exception of TLR3, all TLRs activate the MyD88-dependent pathway. TLR4 activates both the MyD88-dependent and TRIF-dependent pathways. TLR3 exclusively activates the TRIF-dependent pathway. This diagram was generated using BioRender.

Upon the recognition of TLR ligands, TLR signaling will be triggered to induce an intracellular cascade and eventually induce master transcriptional factors such as NF-κB to induce pro-inflammatory cytokines, chemokines, and other effector factors. The activation of TLR signaling is not only inducing innate immune response but also critical as a bridge for an adaptive immune response (14). Based on the localization, TLRs can be divided into two categories. The cell surface TLRs include TLR1/2, TLR2/6, TLR4, TLR5, and TLR10, which mainly recognize bacterial molecular patterns; the endosomal TLRs include TLR3, TLR7, TLR8, and TLR9, in which TLR3 recognizes double-stranded RNA; TLR7 and TLR8 recognize single-stranded RNA and TLR9 recognizes unmethylated CpG motifs in DNA (**Figure 1**). Increasing evidence shows that TLRs play a critical role not only in the protection and clearing of pathogens but also in other immune-related diseases including autoimmune diseases and cancer. Accumulating evidence suggests that TLRs play a critical role in regulating cancer development by controlling the tumor immune microenvironment and influencing treatment efficacy. We are among the first research groups to demonstrate that certain TLRs, such as TLR5, are functional not only in traditional innate immune cells like macrophages and dendritic cells but also in breast cancer epithelial cells (15). Here we summarize the recent advances regarding the role of TLRs in breast cancer pathogenesis and potential implications for therapeutic interventions.

## 3 Toll-like receptors in breast cancer

### 3.1 Synopsis of breast cancer and the tumor immune microenvironment

Breast cancer has remained an arduous challenge to women’s health worldwide; local and systemic immunity plays a critical role in breast cancer development and the efficacy of treatments. The tumor microenvironment (TME) is the center that contains or helps spread the tumor and determine the outcome of different treatments; the crosstalk between breast cancer and immune cells shapes the tumor immune microenvironment (TIME) (16, 17).

To protect the host, the immune system responds to breast cancer inducing anti-tumor activity, in which many kinds of cells are involved, including antigen-presenting cells (APCs) (such as macrophages and dendritic cells), natural killer cells, and cytotoxic T lymphocytes (18). Antitumor activity is the main process by which APCs recognize tumor antigens to present to T lymphocytes which release cytotoxic molecules to induce apoptosis (19, 20).

However, tumor cells have developed a sophisticated mechanism to suppress or evade the anti-tumor immune response by creating an immunosuppressive microenvironment. In the immunosuppressive microenvironment, tumor cells induce the accumulation of myeloid-derived suppressor cells (MDSCs) and T regulatory cells (Tregs), as well as via the axis of programmed cell death ligand 1 (PD-L1) on the tumor cells/PD-1 on the T cells. The PD-L1/PD-1 axis induces T cell exhaustion and impairs T cell cytotoxic function. MDSCs, Tregs, and PD-L1/PD-1 altogether dampen anti-tumor immunity (21).

The interplay between tumor cells and immune cells becomes further complicated due to the heterogeneity of breast cancer, and different subtypes of breast cancer display quite different immune cell profiling and different responses to different treatments. For example, triple-negative breast cancer (TNBC) does not respond well to traditional treatment such as chemotherapy and radiotherapy but is more responsive to immune checkpoint inhibitors compared to other subtypes due to the TNBC with higher levels of tumor-infiltrating lymphocytes (22).

In recent years, cancer immunotherapy in breast cancer treatment has become more attractive. Immune checkpoint inhibitors such as PD-L1/PD-1 antibody showed remarkable results in some breast cancer patients. Additionally, other strategies for enhancing anti-tumor immunity have also been extensively explored recently, including chimeric antigen receptor T cells (CAR-T), cancer vaccines, and the combinations of immune checkpoint inhibitors with other conventional therapies (23, 24). CAR-T cell therapy utilizes the patient’s own T-cells by genetically modifying them to produce chimeric antigen receptors on their surface. These CARs are designed to recognize and bind to specific proteins found in cancer cells. CAR-T cell therapy has demonstrated great success in the treatment of lymphoma and leukemia; however, it can cause severe side effects that require close monitoring and management. In addition, it remains largely ineffective for solid tumors. All these strategies have been shown to be beneficial only for some breast cancer patients. A novel strategy to improve breast cancer treatment is still desperately needed, and targeting innate immunity such as Toll-like receptors holds great promise for that. A better understanding of the interplay between tumor cells and immune cells within TME by viewing from the lens of innate immunity is essential for the development of more effective and personalized treatment for breast cancer.

### 3.2 The variable roles of TLRs in breast cancer cells and the TME

**Figure 2:**
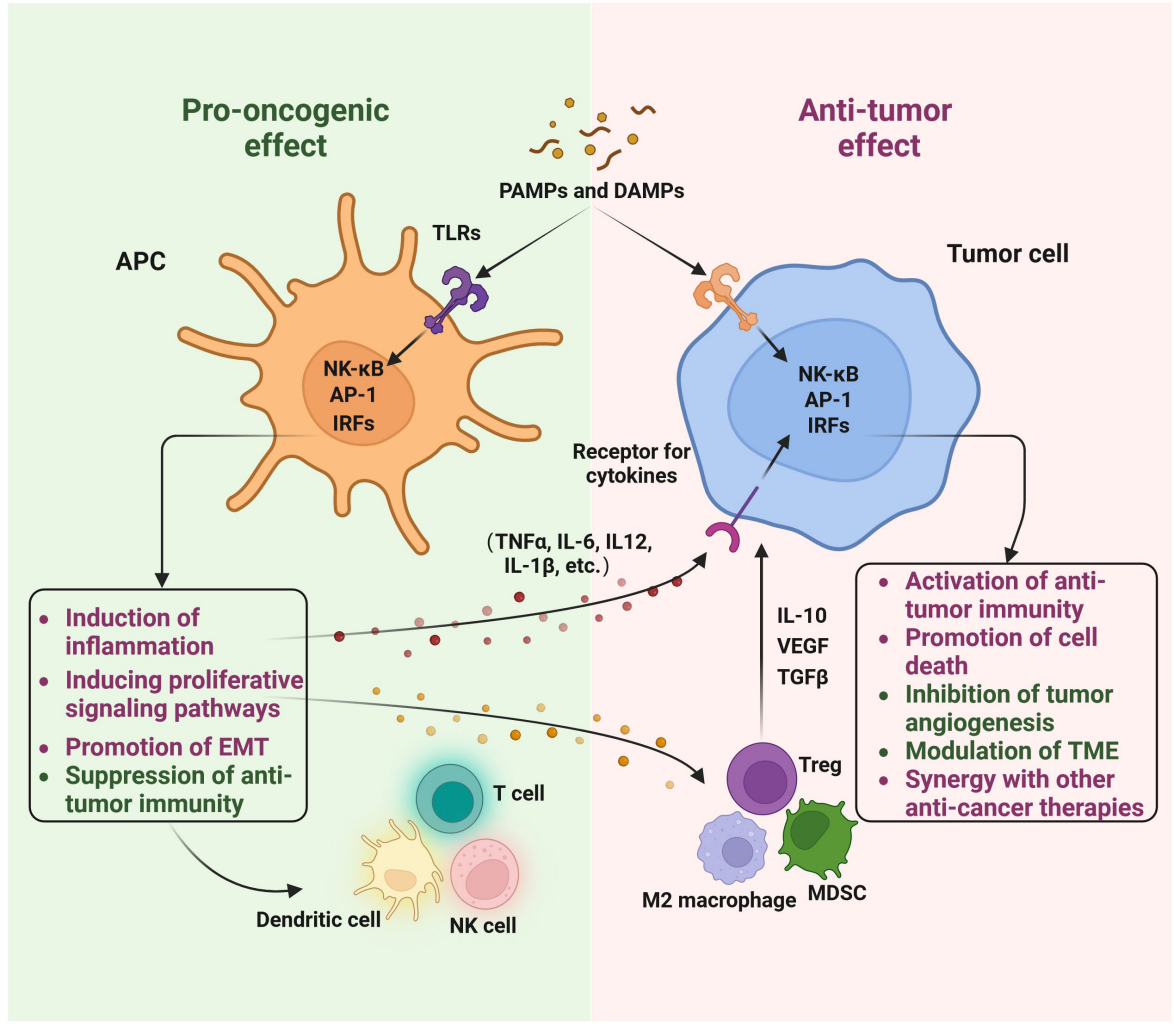
The role of Toll-like receptors in breast cancer: a double-edged sword. The involvement of Toll-like receptors (TLRs) in breast cancer is significant. Both innate immune cells and breast cancer cells recognize pathogen-associated molecular patterns (PAMPs) and damage-associated molecular patterns (DAMPs) to initiate TLR signaling. This activation of TLRs can lead to pro-oncogenic effects, such as triggering inflammation, activating proliferative signaling pathways, promoting epithelial-mesenchymal transition (EMT), and suppressing anti-tumor immunity. Conversely, TLR activation can also exert anti-tumor effects, including the activation of anti-tumor immunity, induction of cell death, inhibition of tumor angiogenesis, modulation of the tumor microenvironment (TME), and synergy with other anti-cancer therapies. This diagram was generated using BioRender.

**Figure 3:**
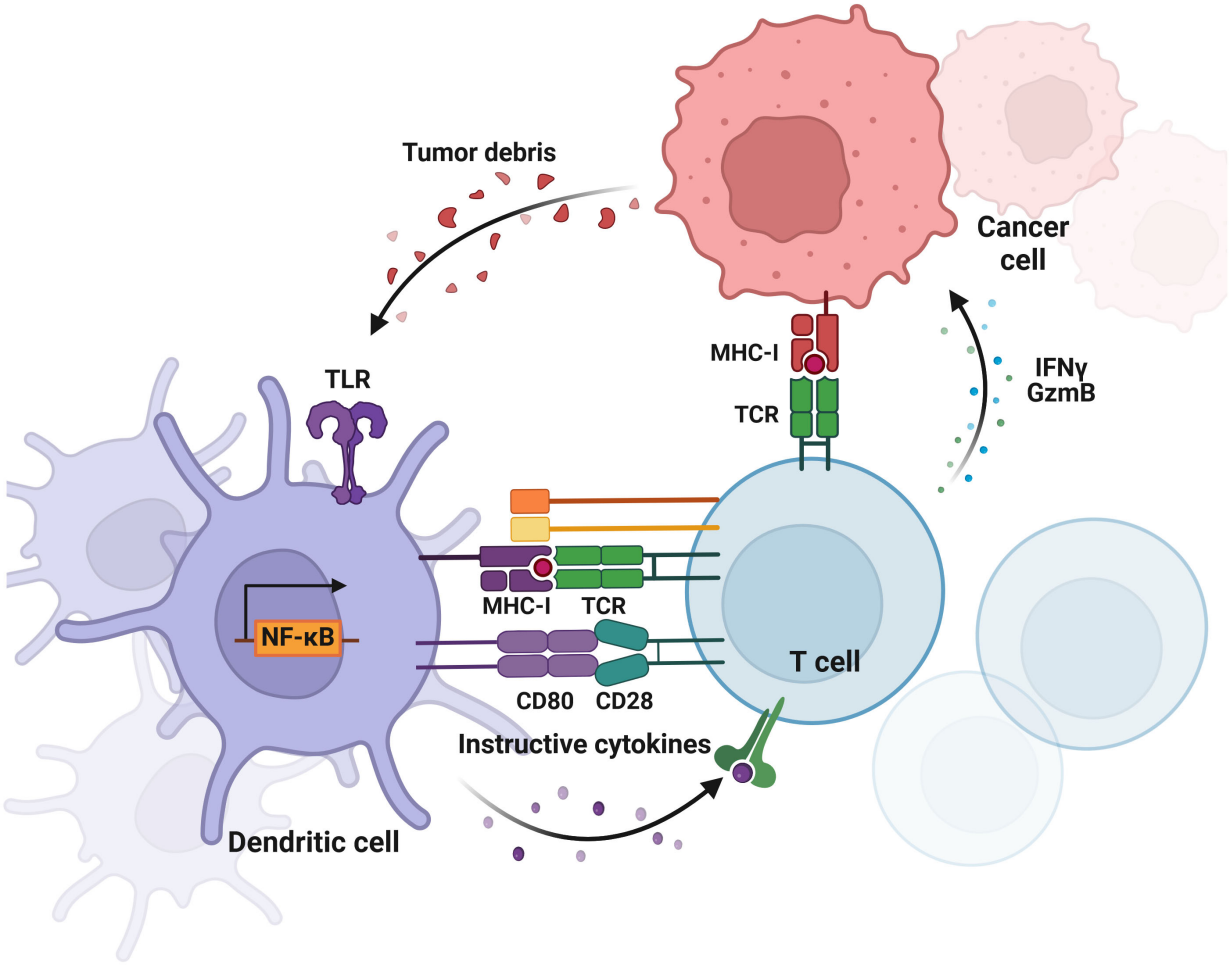
Crosstalk between tumor cells and DCs in breast cancer. The diagram illustrates the bidirectional interactions where tumor cells release signals that influence DC function and phenotype, while DCs modulate tumor growth and immune response through T cell activation. This diagram was generated using BioRender.

TLRs were originally uncovered as the long-time-seeking key receptors in innate immune systems, which recognize microbial molecular patterns such as LPS to activate intracellular signaling cascades in antigen-presenting cells (APCs) (**Figures 2**, **3**). Soon after the discovery of TLRs in APCs, accumulating reports, including from us, showed that TLRs are expressed and activated in cancer epithelial cells (15, 25), indicating that TLRs might play a role in tumor development. However, further studies showed that the activation of different TLRs in different cancers can play an opposite role (**Tables 1**, **2**) although different TLRs share similar TLR signaling pathways (**Figure 2**), and the underlying mechanism is still not well understood. The activation of TLRs in both tumor cells and immune cells, inducing sequential or synergetic signaling, might play a role in the diverse consequences of TLR activation in breast cancer (**Tables 1**, **2**).

**Table 1:** Pro-tumorigenic roles of TLRs in breast cancer.

TLRs	Main Cell Types involved	Pro-tumorigenic Activity (references)	Refs
TLR2	Breast cancer cells (MDA-MB-231 cells) Cancer stem cells	Promoting invasiveness and adhesiveness Promoting breast cancer progression and resistance to chemotherapy	(26, 27) (28)
TLR3	Endothelial cells	Driving metastasis with TLR3-SLITs axis	(29)
TLR4	Breast cancer cells (MDA-MB-231 cells) Breast cancer cells (MDA-MB-231 and MCF7 cells)	Inducing proliferation and mediating the secretion of pro-inflammatory cytokines (e.g., IL-6 and IL-8) Promoting tumor growth and metastasis	(30) (31)
TLR7	Dendritic cells	Impact on survival	(32)
TLR9	Human breast cancer cells	Promoting invasion	(33, 34)

**Table 2:** Anti-tumorigenic roles of TLRs in breast cancer.

TLRs	Main Cell Types involved	Anti-tumorigenic Activity	Refs
TLR2	NK cells dendritic cells (DC)	Enhancing the antitumor effect Eliciting anti-tumor immunity	(35) (36)
TLR3	Human breast cancer cells	inducing apoptosis	(37, 38)
TLR4	Dendritic cells	Inducing anti-tumor immunity	(39)
TLR5	Human breast cancer cells	Inhibiting breast cancer cell proliferation and tumor growth	(24)
TLR7	Plasmacytoid DC myeloid-derived suppressor cells (MDSC)	Favoring prognosis Anti-tumor immunity to activate T cell function	(40) (41)
TLR8	Human breast cancer cells Dendritic cells	Preventing tumor-induced T-cell senescence Activating cytotoxic T lymphocytes	(42) (36)
TLR9	Triple-negative breast cancer cells Fibroblast-like cells	Favoring prognosis Low probability of metastasis	(43) (44)

Based on the localization, TLRs can be expressed on the cell surface or endosomal compartment as mentioned before. Based on the phylogenetic tree analysis by our group and others (45, 46), TLRs can be divided into five subfamilies, including 1) TLR1/2/6/10; 2) TLR3; 3) TLR4; 4) TLR5; 5) TLR7/8/9. Among human TLRs, TLR2 functions via a heterodimer with TLR1 or TLR6, the specific ligand for TLR10 is not well defined yet, and other TLRs function mainly through homodimers. While the exact ligands for TLR10 are not fully elucidated, TLR10 may function like TLR1 or TLR6 by heterodimerizing with TLR2 (47). Given the lack of a clear mechanistic understanding, we will not discuss the potential role of TLR10 in breast cancer in this review article.

#### 3.2.1 TLR2 subfamily in breast cancer

TLR2 holds a unique position within the TLR family, typically necessitating heterodimerization with either TLR1 or TLR6 to initiate the MyD88-dependent TLR signaling pathway. These heterodimerizations enhance the diversity of ligand recognition. Numerous pieces of evidence underscore the pivotal role of TLR2 in breast cancer.

Analysis of TCGA data reveals significant involvement of TLR2, TLR1, and TLR6 in breast cancer. However, the role of TLR2 in this context appears intricate. Depending on the specific receptor combination, TLR2 may exhibit varying roles. In breast cancer, TLR2 has been observed to both promote (**Table 1**) and inhibit tumor growth (**Table 2**), highlighting its dual functionality and pleiotropic roles in tumorigenesis. The precise mechanism underlying this discrepancy remains elusive and warrants further investigation.

##### 3.2.1.1 The pro-tumorigenic role of TLR2

Multiple studies have highlighted the adverse impact of TLR2 expression and activation on breast cancer outcomes. For instance, analyses of human breast cancer samples consistently reveal elevated TLR2 expression, which correlates with poor overall survival and resistance to endocrine therapy in the luminal B subtype of breast cancer (26). Xie et al. observed a significant upregulation of TLR2 expression in highly metastatic MDA-MB-231 cells compared to poorly metastatic counterparts and non-transformed breast cells (48). Their findings elucidated the pivotal role of TLR2 activation by infectious bacterial peptidoglycan (PGN) in bolstering breast cancer cell invasiveness (48), unveiling a novel link between infectious bacteria and cancer cells with potential therapeutic implications for antibiotic intervention in cancer treatment. Notably, polymorphisms in the TLR2 gene, such as rs5743708, may predispose individuals to breast cancer (28).

Furthermore, Lorenzo et al. characterized TLR2WT and TLR2KO autochthonous mammary cancer mouse models, uncovering a pro-tumorigenic role of TLR2 in driving breast cancer progression and fostering resistance to chemotherapy (49). Consistent with these findings, the deletion of TLR2 in a mouse spontaneous development of breast cancer model (MMTV-Wnt1 transgenic mice) resulted in reduced breast tumor growth and metastasis (27). Moreover, TLR2 was found to promote cancer stem cell self-renewal, as evidenced by studies utilizing TLR2 knockout mouse models, which demonstrated that TLR2 is crucial for cancer stem cell maintenance and the induction of regulatory T cells (27). Additionally, it has been shown that the release of HMGB1 induced by doxorubicin activates TLR2 signaling in cancer cells, contributing to a chemotherapy-resistant phenotype (49). Furthermore, the inhibition of TLR2 has been shown to reduce tumor growth and enhance the efficacy of doxorubicin without adversely affecting the host immune system *in vivo* (50).

##### 3.2.1.2 The tumor-suppressive role of TLR2

Conversely, TLR2 has recently emerged as a pivotal player in inhibiting tumor growth in breast cancer. Upon activation, TLR2 initiates a cascade of anti-tumor mechanisms, including cytokine production and the activation of immune cells, particularly promoting macrophage differentiation towards the M1 phenotype. These concerted efforts effectively suppress tumor progression (35). Moreover, polysaccharide krestin (PSK), the natural ligand of TLR2, stimulates TLR2 and induces NK cell-mediated antitumor activity in HER2+ breast cancer cells (36). Green et al. investigated TLR expression on dendritic cells and unveiled the crucial role of TLR2 for triggering anti-tumor immunity, in metastatic breast cancer patients with circulating tumor cells (51). Propranolol, a β-adrenergic signaling antagonist, demonstrates palliative effects on breast cancer progression and survival, primarily through TLR2-directed immunomodulation, such as altering the polarization of macrophages, and increasing the activation of cytotoxic T cells and NK cells (52).

In murine models, TLR2 activation has demonstrated the ability to suppress breast tumor growth and metastasis. These anti-tumor effects are mediated through the inhibition of the AKT/mTOR signaling pathway, which governs cell growth and survival (53). The capacity of TLR2 to thwart oncogenic AKT/mTOR signaling suggests that TLR2 agonists could be explored as potential breast cancer therapeutics, leveraging the innate immune response against tumors.

Furthermore, Amirfakhri et al. demonstrated that conditioned media from cultured MCF7 breast cancer cells upregulate TLR2 expression on NB4 neutrophil-like cell line, suggesting a potential role of breast cancer cells in modulating TLR2 activity within the tumor microenvironment (54). Additionally, Khosravi et al. explored the impact of exercise on monocyte function in breast cancer survivors, revealing that acute aerobic exercise downregulates TLR2 and TLR4 expression on monocytes and attenuates intracellular pro-inflammatory cytokine production (55), although the precise molecular mechanisms remain elusive.

#### 3.2.2 TLR3 in breast cancer

TLR3 plays a multifaceted and intricate role in breast cancer pathogenesis. As a vital component of the innate immune system, it detects exogenous double-stranded RNA (dsRNA), a marker often associated with a viral infection. Unlike other TLRs, TLR3 exclusively activates the MyD88-independent TIR domain-containing adaptor-inducing IFN-beta (TRIF) pathway.

In breast cancer, TLR3 predominantly serves as a tumor-suppressive factor. The stimulation of TLR3 by its agonist, dsRNA, prompts apoptosis in human breast cancer cells (37). Studies, such as that conducted by Fan et al. have linked a specific single nucleotide polymorphism (SNP) in the TLR3 promoter region (rs5743305) with an increased risk of breast cancer, suggesting the role of TLR3 as a tumor suppressor gene (38, 56). Further investigations revealed that TLR3 activation, particularly by synthetic ligands like poly(I:C), inhibits breast cancer cell growth and induces apoptosis, thus suppressing tumor growth and metastasis in mouse models. These effects are attributed to the induction of apoptosis, inhibition of angiogenesis, and stimulation of anti-tumor immunity involving natural killer cells and cytotoxic T cells through the TLR3-induced IFN-dependent TRAIL pathway (57). TLR3 expression in DCs plays a crucial role in breast cancer. Notably, high expression of TLR3 in triple-negative breast cancer indicates a better prognosis (38), and TLR3-activated cDCs produce IFN- λ, which is linked to a good clinical outcome in breast cancer (58). Consequently, TLR3 ligands, such as poly I:C, are being investigated as potential breast cancer therapies (29).

TLR3 activation in breast cancer cells can also promote tumor growth and metastasis by triggering inflammation, angiogenesis, and immune suppression through pathways such as NF-κB and MAPK, by promoting the expression of pro-inflammatory cytokines, angiogenic factors, and regulatory cytokines (59). Understanding how these pathways regulate these processes in the context of TLR3 activation could provide insights into the complex roles TLR3 plays in breast cancer progression and tumor microenvironment modulation. Tumor cells exhibiting high TLR3 expression correlate with a higher likelihood of metastasis (30). Studies have reported elevated TLR3 expression in breast cancer cells compared to normal cells, with TLR3 activation linked to increased cell proliferation, migration, invasion, and epithelial-mesenchymal transition (EMT) *in vitro*, as well as enhanced tumor growth and metastasis in mouse xenograft models (60).

The context-dependent roles of TLR3 in breast cancer are influenced by various factors including cancer stage, tumor microenvironment, and specific signaling pathways activated. Harnessing the immunostimulatory properties of TLR3 while mitigating its pro-tumorigenic effects holds promise for targeted therapies against breast cancer. For therapeutic potential, research by Huang et al. explores engineered exosomes as an *in-situ* DC-primed vaccine to enhance antitumor immunity in breast cancer. These exosomes, induced by TLR3, demonstrate the ability to induce immunogenic cell death specifically in breast cancer cells, highlighting their potential in therapeutic interventions (61).

#### 3.2.3 TLR4 in breast cancer

TLR4 is the first identified human TLR as a pattern recognition receptor that initiates innate immune responses upon recognizing PAMPs like lipopolysaccharide (LPS). Its role in breast cancer is complex and appears to be context-dependent.

##### 3.2.3.1 Pro-tumorigenic effects

Increased expression of TLR4 has been consistently observed in both breast cancer cells and tissues compared to their normal counterparts (31). Studies have shown that decreased expression of TLR4 inhibits the proliferation of human breast cancer MDA-MB-231 cells and suppresses the secretion of inflammatory cytokines including IL-6 and IL-8 (31).

TLR4 activation, whether by lipopolysaccharides (LPS) or endogenous ligands, has been found to significantly promote various cancerous behaviors in breast cancer cells *in vitro*. These include enhanced proliferation, survival, migration, and invasion. Such effects are primarily mediated through pathways involving NF-κB, PI3K/AKT, and other oncogenic signaling pathways. Furthermore, TLR4 signaling induces properties akin to cancer stem cells and facilitates epithelial-mesenchymal transition (EMT), a process implicated in cancer metastasis. The involvement of TLR4 extends to shaping an immunosuppressive microenvironment within tumors by promoting the generation of myeloid-derived suppressor cells. Notably, the expression of TLR4 in the ductal epithelial cells of the breast tumor microenvironment correlates with the invasiveness of the tumor, indicating its potential role in driving breast cancer progression (62). Chronic inflammation is a well-established risk factor for cancer, including breast cancer. TLR4 signaling exacerbates this inflammatory milieu within the tumor microenvironment by stimulating the production of pro-inflammatory cytokines and chemokines, thereby fostering tumor growth and metastasis. Moreover, TLR4 activation directly influences the behavior of breast cancer cells, promoting their proliferation, survival, and resistance to apoptosis, which collectively contribute to tumor progression.

Interestingly, TLR4 signaling appears to intersect with estrogen receptor signaling pathways in breast cancer. Studies indicate that estrogen can upregulate TLR4 expression, and conversely, activation of TLR4 enhances estrogen-mediated signaling, potentially fueling estrogen-driven breast cancer growth. Additionally, TLR4 activation in breast cancer cells modulates immune responses, promoting immune evasion through the secretion of immunosuppressive factors and inhibition of anti-tumor immune responses. Clinically, TLR4 overexpression serves as an independent predictor of poor disease-free survival in breast cancer patients (39). TLR4 has emerged as a promising therapeutic target in breast cancer due to its role in promoting tumor growth and progression. Targeting TLR4 signaling pathways, either directly or indirectly, may offer novel therapeutic strategies for breast cancer treatment (63, 64). For instance, the level of TLR4 expression can serve as a novel determinant of the response to paclitaxel in breast cancer (63). Additionally, recent findings indicate that small extracellular vesicles derived from *Fusobacterium nucleatum* facilitate tumor growth and metastasis via TLR4 in breast cancer (65). The literature underscores the significant role of TLR4 in breast cancer development and progression, highlighting its potential as a therapeutic target in breast cancer therapy. Further research is imperative to comprehensively understand the mechanisms underlying TLR4 involvement in breast cancer and to systematically explore its therapeutic potential *in vivo*.

##### 3.2.3.2 Anti-tumorigenic effects

Several studies indicate that TLR4 activation may impede breast cancer cell growth and prompt apoptosis. In specific scenarios, TLR4 triggers anti-tumor immune responses by activating dendritic cells and cytotoxic T lymphocytes (66, 67). Furthermore, TLR4 agonists have demonstrated anti-metastatic effects in specific breast cancer models (66). The involvement of TLR4 in breast cancer cell signaling has been highlighted, particularly focusing on the role of LPS/TLR4 signaling in breast cancer development. There are promising prospects for targeting this axis in treatment (66). TLR4 activation on immune cells, such as dendritic cells, can enhance anti-tumor immunity by promoting the expression of pro-inflammatory mediators and cytokines like IFN-γ (67). Furthermore, TLR4 on DCs presents tumor antigens from dying cancers to activate cytotoxic T cells for fighting cancer (32).

The divergent roles of TLR4 are influenced by factors such as TLR4 ligand dose, tumor subtype, immune status, and the tumor microenvironment. In general, constitutive TLR4 signaling tends to support pro-tumorigenic functions, whereas regulated or therapeutic TLR4 activation may promote anti-tumor immunity. Ongoing research endeavors seek to further investigate the complex role of TLR4 and assess whether targeting TLR4 could be a viable therapeutic strategy. This approach may involve combining TLR4 targeting with existing breast cancer treatments that modulate the tumor microenvironment and enhance anti-tumor immunity.

#### 3.2.4 TLR5 in breast cancer

TLR5 is a member of the TLR family that recognizes bacterial flagellin, a structural component of flagella in bacteria. Research suggests that TLR5 expression may vary among different subtypes of breast cancer and could have diverse effects on tumor progression and the immune response to cancer. However, TLR5 is most likely to play an anti-tumor role in breast cancer development. The TCGA data show that TLR5 high expressed indicates a favorable prognosis in breast cancer patients.

We were among the first groups to discover that TLR5 functions not only in innate immune cells but also in cancer epithelial cells (15). We found that TLR5 is highly expressed in breast carcinomas and is overly responsive in breast cancer cells (15, 40). Activation of TLR5 by flagellin has been shown to suppress cell proliferation and tumor growth in breast cancer cells, indicating that TLR5 may serve as a novel therapeutic target for human breast cancer therapy (15). Additionally, TLR5 agonist flagellin has been found to inhibit the cell state of activation and induce autophagy, and the autophagy protein MAP1S (Microtubule Associated Protein 1S) has been shown to regulate the flagellin/TLR5 signaling pathway in breast cancer cells (68, 69). Furthermore, TLR5 overexpression has been associated with lymph node metastasis and cancer grade in breast carcinomas, and TLR5 SNP rs5744168 has been found to be associated with sporadic breast cancer occurrence (41). Finally, the importance of TLR5 in mediating anti-tumor responses is being explored, with evidence that TLR5 agonists enhance anti-tumor immunity and overcome resistance to immune checkpoint therapy (70).

However, the roles of TLR5 in breast cancer may be attributed to the complex interplay between the tumor cells, the tumor microenvironment, and the host immune system. TLR5 signaling can stimulate the production of pro-inflammatory cytokines and chemokines that create a tumor-promoting microenvironment. The activation of TLR5 on dendritic cells can promote the presentation of tumor-associated antigens to T cells, enhancing anti-tumor immunity and overcoming resistance to immune checkpoint therapy in a syngeneic breast cancer model (70). Furthermore, TLR5 expression and activation may interact with other signaling pathways implicated in breast cancer, such as the NF-κB pathway, further influencing tumor behavior. Additionally, the specific breast cancer subtype and the stage of the disease may also influence the outcome of TLR5 activation. Further research is needed to fully elucidate the mechanisms by which TLR5 signaling can inhibit breast cancer progression and to explore the potential therapeutic applications of TLR5 agonists or antagonists in combination with other immunotherapeutic approaches.

#### 3.2.5 TLR7 in breast cancer

TLR7 is an endosomal TLR that recognizes single-stranded RNA (ssRNA), which can be derived from viruses or endogenous sources. In recent years, research has uncovered the potential significance of TLR7 in breast cancer, with evidence suggesting both pro-tumorigenic and anti-tumor effects.

##### 3.2.5.1 Pro-tumorigenic effects

TLR7 activation by ssRNA or synthetic agonists has been shown to promote breast cancer cell proliferation, migration, and invasion *in vitro*. These effects are thought to be mediated through the activation of NF-κB and other oncogenic signaling pathways, leading to the production of pro-inflammatory cytokines and chemokines that can support tumor progression (71). Shi et al. analyzed data from the Cancer Genome Atlas (TCGA) database and found that higher expression levels of TLR7 were associated with worse prognosis in breast cancer patients (71).

##### 3.2.5.2 Anti-tumor effects

TLR7 stimulation has been shown to activate dendritic cells and promote the cross-presentation of tumor-associated antigens, leading to the activation of cytotoxic T lymphocytes and anti-tumor immunity. The anti-tumor effects of TLR7 activation may depend on factors such as the dose of the agonist, the tumor microenvironment, and the breast cancer subtype. Mercier et al. reported that tumor-associated plasmacytoid dendritic cells (pDC) in human primary breast tumors are associated with poor outcomes, which can be reversed by TLR7 ligand treatment (42). Yin et al. found that the TLR7/8 agonist R848 exhibits antitumoral effects in a breast cancer model (72). Hosoya et al. demonstrated the induction of oligoclonal CD8 T cell responses against pulmonary metastatic cancer by a phospholipid-conjugated TLR7 agonist (73), highlighting the importance of TLR7 agonists in enhancing antitumor immunity against breast cancer. Safarzadeh et al. showed that STAT3 silencing and TLR7/8 pathway activation could repolarize and suppress myeloid-derived suppressor cells to activate T-cell function in breast cancer patients (33).

Several studies have explored the potential therapeutic applications of TLR7 agonists or antagonists in combination with other immunotherapeutic approaches for breast cancer treatment (34, 74). However, further research is needed to fully understand the mechanisms by which TLR7 signaling can either promote or inhibit breast cancer progression and to optimize the potential therapeutic strategies targeting this pathway. Ayala et al. showed that dual activation of TLR7 and TLR9 impaired the efficacy of antitumor vaccines in murine models of metastatic breast cancer (34). Furthermore, Huang et al. developed a nanoparticle-integrated dissolving microneedle system for the co-delivery of R848 and anti-PD-1 antibody to reverse the immunosuppressive microenvironment of triple-negative breast cancer (74).

Overall, while TLR7 holds promise as a target for breast cancer therapy, further research is needed to fully understand its role in breast cancer progression and its potential as a therapeutic target. Several studies have explored the potential therapeutic applications of TLR7 agonists or antagonists in combination with other immunotherapeutic approaches for breast cancer treatment (34, 74). However, further research is needed to fully understand the mechanisms by which TLR7 signaling can either promote or inhibit breast cancer progression and to optimize the potential therapeutic strategies targeting this pathway.

#### 3.2.6 TLR8 in breast cancer

TLR8, like TLR7, is an endosomal receptor that recognizes single-stranded RNA (ssRNA) molecules. TLR8 is primarily known for its role in recognizing viral RNA and activating immune responses against viral infections. Its role in breast cancer has been less extensively studied compared to some other TLRs, but emerging evidence suggests that it may contribute to breast cancer progression and modulate anti-tumor immunity (75). Increased TLR8 expression has been observed in breast cancer cell lines and tumor tissues compared to normal breast cells and tissues (71). Roychowdhury et al. explored the landscape of TLR expression in the tumor microenvironment of triple-negative breast cancer (TNBC), identifying distinct roles of TLR4 and TLR8 in the maintenance of the tumor-immune microenvironment (43). TLR8 stimulation may activate dendritic cells and promote the presentation of tumor-associated antigens, leading to the activation of cytotoxic T lymphocytes and anti-tumor immune responses (51). In a study by Zhou et al., nanoparticles loaded with a TLR7 and TLR8 agonist were used to treat metastatic breast cancer, combining anti-angiogenesis and immune activation for therapeutic benefits (44).

It is important to note that the research on the role of TLR8 in breast cancer is relatively limited compared to some other TLRs, and further studies are needed to fully elucidate its mechanisms of action and potential therapeutic implications. Additionally, TLR8 is closely related to TLR7, and their functions may be interconnected. Therefore, the interplay between TLR8 and TLR7, as well as their combined effects on breast cancer progression and immunity, require further investigation.

#### 3.2.7 TLR9 in breast cancer

TLR9 is another endosomal TLR that recognizes unmethylated CpG DNA, which is commonly found in bacterial and viral genomes, as well as in certain endogenous nucleic acids released from dying cells.

In certain contexts, TLR9 signaling has been associated with tumor-promoting effects in breast cancer. Activation of TLR9 signaling pathways can promote tumor cell proliferation, survival, and invasion by inducing the production of pro-inflammatory cytokines, chemokines, and growth factors. TLR9 activation may also contribute to the establishment of an immunosuppressive tumor microenvironment, thereby facilitating tumor progression. For example, TLR9 has been found to be expressed in human breast cancer cells and clinical breast cancer samples and TLR9 agonists can promote cellular invasion by increasing matrix metalloproteinase activity in breast cancer cells (76). Furthermore, CpG oligonucleotides, known to stimulate TLR9, have been shown to induce invasion in breast cancer cells (77). The expression of TLR9 has been associated with poorly differentiated tumors in breast cancer specimens, suggesting a potential role in cancer progression (78). Moreover, the expression of TLR9 has been investigated in relation to lymph node metastasis in breast cancer, with positive TLR9 status potentially serving as an indicator of poor prognosis (79).

Conversely, there is evidence suggesting that TLR9 activation may have anti-tumor effects in breast cancer. The expression and functional role of TLR9 in breast cancer may vary across different breast cancer subtypes. Some studies have suggested that TLR9 expression may be associated with specific molecular subtypes of breast cancer, such as triple-negative breast cancer. Low TLR9 expression has been linked to an aggressive subtype of triple-negative breast cancer and poor disease-specific survival in patients with triple-negative breast cancer, indicating that TLR9 expression is favorable for the patient prognosis with this type of breast cancer (80). Breast cancer patients with high TLR9 expression by fibroblast-like cells were associated with low probability of metastasis (81). The activation of TLRs, including TLR9, in plasmacytoid dendritic cells can inhibit breast cancer cell growth by directly killing tumor cells and activating NK cells and CD8+ T cells (82). The anti-tumor effects of TLR9 activation may depend on factors such as the dose and type of the ligand, the tumor microenvironment, and the breast cancer subtype.

In summary, TLR9 signaling in breast cancer is complex and context-dependent, exerting both pro-oncogenic and anti-tumor effects. Further research is needed to elucidate the mechanisms underlying TLR9 signaling in breast cancer pathogenesis and to explore its potential as a therapeutic target for breast cancer treatment.

## 4 Targeting toll-like receptors for improving breast cancer immunotherapy

Targeting Toll-like receptors (TLRs) represents a promising approach to enhance the effectiveness of immunotherapies for breast cancer. By modulating TLR signaling, researchers aim to stimulate anti-tumor immune responses and overcome the immunosuppressive tumor microenvironment.

TLR signaling in tumor-associated macrophages (TAMs) can influence their polarization and function within the tumor microenvironment. Modulating TLR signaling in TAMs towards an anti-tumor M1 phenotype may lead to the secretion of pro-inflammatory cytokines and the recruitment of effector T cells, thereby enhancing anti-tumor immunity in breast cancer. Wei et al. developed polymer micelles to deliver TLR7 agonist R837 and doxorubicin to TAMs and tumor cells for enhanced chemo-immunotherapy against breast cancer, showcasing a novel approach in nanomedicine development (83). TLR agonists can be used to reshape the immunosuppressive breast tumor microenvironment by stimulating the recruitment and activation of immune effector cells, such as dendritic cells, natural killer cells, and cytotoxic T lymphocytes. Additionally, TLR signaling can modulate the function of immunosuppressive cells, like myeloid-derived suppressor cells and regulatory T cells, reducing their inhibitory effects on anti-tumor immunity in breast cancer. Gatti-Mays et al. highlighted the potential of leveraging antitumor immunity in breast cancer to overcome immunologic resistance, shedding light on the unique aspects of the tumor microenvironment (84).

TLR agonists can be employed as immunoadjuvants in breast cancer vaccines or adoptive cell therapies to boost anti-tumor immune responses. By activating TLRs on antigen-presenting cells (APCs), these agonists can promote antigen presentation, co-stimulatory molecule expression, and cytokine production, leading to stronger T-cell priming against tumor-associated antigens. Examples include TLR3 agonist poly(I:C), TLR7/8 agonists like imiquimod and resiquimod, and TLR9 agonists like CpG oligodeoxynucleotides (85). TLR agonists are being evaluated in combination with immune checkpoint inhibitors (e.g., anti-PD-1, anti-PD-L1, anti-CTLA-4) for breast cancer treatment. TLR activation can prime the immune system and promote T cell activation, while checkpoint inhibitors alleviate the suppressive mechanisms that limit anti-tumor immunity (86). This synergistic approach aims to overcome resistance mechanisms and improve clinical responses in breast cancer patients (87, 88). Given the heterogeneity of breast cancer subtypes and the complex interplay between TLRs and the immune system, personalized approaches may be necessary to optimize TLR-targeted immunotherapies. Factors like TLR expression profiles, tumor mutational burden, immune cell infiltration, and breast cancer subtype can guide the selection of appropriate TLR agonists and combination strategies for individual patients (89).

Overall, the role of TLRs in breast cancer immunotherapy is multifaceted, and further research is needed to fully elucidate their functions and therapeutic potential in this context. Continued efforts to develop novel TLR-targeted immunotherapies and to better understand the complex interplay between TLR signaling and the tumor microenvironment will be crucial for advancing breast cancer treatment strategies.

## 5 Conclusion and outlook

Overall, the exploration of TLRs in the context of breast cancer has unveiled a complex yet promising avenue for understanding and treating this devastating disease. TLRs, key players in the immune system’s arsenal, exhibit a dual nature in their interaction with cancer, capable of both promoting and inhibiting tumor progression. This duality underscores the intricate interplay between the immune system and tumor cells, revealing that TLRs can influence various facets of the cancer-immunity cycle, including cell proliferation, survival, metastasis, and the immune microenvironment. While some TLRs, such as TLR2 and TLR4, have shown both promotive and suppressive effects on tumor growth, others like TLR3 and TLR5 offer promising avenues for therapeutic intervention due to their more defined roles in enhancing anti-tumor immunity. The diversity in TLR functions underscores the complexity of targeting these receptors for cancer therapy, necessitating a detailed understanding of their mechanisms of action.

Looking forward, the prospect of TLR-targeted therapies in breast cancer appears promising but demands a concerted endeavor to unravel the intricate dynamics of TLR signaling within the tumor microenvironment. The potential of TLR agonists and antagonists, especially when combined with existing treatments such as chemotherapy, radiation therapy, and immune checkpoint inhibitors, unveils novel avenues for enhancing patient outcomes. For instance, the capacity of TLR3 ligands to augment the effectiveness of breast cancer treatment has been examined in multiple clinical trials (29). However, realizing this potential depends on surmounting several hurdles, including precisely deciphering the dual roles of TLRs in cancer progression, identifying specific TLR ligands and their impacts on both tumor cells and immune cells, and elucidating the broader ramifications of TLR modulation on the immune system. Examining the molecular mechanisms underlying the bifunctionality of TLRs in cancer is imperative. Advanced methodologies like single-cell sequencing and *in vivo* modeling can illuminate the context-dependent roles of TLRs in the tumor microenvironment. Furthermore, expanded clinical trials are essential to assess the efficacy and safety of TLR-targeted therapies. These trials should strive to pinpoint biomarkers for patient selection and optimize treatment protocols based on tumor subtype, stage, and the individual patient’s immune profile. We are at the cusp of developing more efficacious and personalized treatment strategies, which take into account the genetic, molecular, and immunological landscape of individual tumors for maximizing their therapeutic efficacy.

In conclusion, TLRs offer fertile ground for research and therapeutic innovation in breast cancer. The dualistic nature of their role in cancer biology presents both challenges and opportunities. By deepening our comprehension of TLR signaling pathways and harnessing their potential to modulate the immune response, we can unlock novel therapeutic pathways. These endeavors hold the promise of augmenting treatment outcomes, mitigating therapy resistance, and ultimately enhancing the survival and quality of life for breast cancer patients.

## Author contributions

JZ: Writing – original draft, Writing – review & editing. LZ: Writing – original draft, Writing – review & editing. SL: Writing – original draft, Writing – review & editing. DD: Writing – review & editing. DZ: Writing – original draft, Writing – review & editing, Conceptualization, Formal Analysis, Funding acquisition.

## Glossary

**Table:**

TLRs	Toll-like receptors
PRRs	pattern recognition receptors
PAMPs	pathogen-associated molecular patterns
DAMPs	damage-associated molecular patterns
PD-1	programmed cell death protein 1
CTLA-4	cytotoxic T-lymphocyte-associated antigen 4
LPS	lipopolysaccharide
TME	tumor microenvironment
TIME	tumor immune microenvironment
APCs	antigen-presenting cells
MDSCs	myeloid-derived suppressor cells
Tregs	T regulatory cells
TNBC	triple-negative breast cancer
CAR-T	chimeric antigen receptor T cells
MyD88	Myeloid differentiation primary response 88
TRIF	TIR domain-containing adaptor-inducing IFN-beta
TCGA	The Cancer Genome Atlas
WT	wildtype
KO	knockout
PGN	peptidoglycan
HMGB1	high mobility group box 1
SNP	single nucleotide polymorphism
EMT	epithelial-mesenchymal transition
DCs	dendritic cells
pDC	plasmacytoid dendritic cells
ssRNA	single-stranded RNA dsRNA, double-stranded RNA

## References

[1] Janeway CA Jr. Pillars article: approaching the asymptote? Evolution and revolution in immunology. Cold spring harb symp quant biol. 1989. 54: 1-13. J Immunol. (2013) 191:4475–87. 24141854

[2] Matzinger P. Tolerance, danger, and the extended family. Annu Rev Immunol. (1994) 12:991–1045. doi: 10.1146/annurev.iy.12.040194.005015 8011301

[3] Rakoff-Nahoum S Medzhitov R. Toll-like receptors and cancer. Nat Rev Cancer. (2009) 9:57–63. doi: 10.1038/nrc2541 19052556

[4] Giaquinto AN Sung H Miller KD Kramer JL Newman LA Minihan A. Breast cancer statistics, 2022. CA Cancer J Clin. (2022) 72:524–41. doi: 10.3322/caac.21754 36190501

[5] Emens LA. Breast cancer immunotherapy: facts and hopes. Clin Cancer Res. (2018) 24:511–20. doi: 10.1158/1078-0432.CCR-16-3001 PMC5796849 28801472

[6] Cui L Wang X Zhang D. TLRs as a promise target along with immune checkpoint against gastric cancer. Front Cell Dev Biol. (2020) 8:611444. doi: 10.3389/fcell.2020.611444 33469538 PMC7813757

[7] Henriques B Mendes F Martins D. Immunotherapy in breast cancer: when, how, and what challenges? Biomedicines. (2021) 9:1–22. doi: 10.3390/biomedicines9111687 PMC8616011 34829916

[8] Medzhitov R Preston-Hurlburt P Janeway CA Jr. A human homologue of the Drosophila Toll protein signals activation of adaptive immunity. Nature. (1997) 388:394–7. doi: 10.1038/41131 9237759

[9] Poltorak A He X Smirnova I Liu MY Van Huffel C Du X. Defective LPS signaling in C3H/HeJ and C57BL/10ScCr mice: mutations in Tlr4 gene. Science. (1998) 282:2085–8. doi: 10.1126/science.282.5396.2085 9851930

[10] Akira S Takeda K Kaisho T. Toll-like receptors: critical proteins linking innate and acquired immunity. Nat Immunol. (2001) 2:675–80. doi: 10.1038/90609 11477402

[11] O'Neill LA Bowie AG. The family of five: TIR-domain-containing adaptors in Toll-like receptor signalling. Nat Rev Immunol. (2007) 7:353–64. doi: 10.1038/nri2079 17457343

[12] Medzhitov R. Toll-like receptors and innate immunity. Nat Rev Immunol. (2001) 1:135–45. doi: 10.1038/35100529 11905821

[13] Matzinger P. Friendly and dangerous signals: is the tissue in control? Nat Immunol. (2007) 8:11–3. doi: 10.1038/ni0107-11 17179963

[14] Kawai T Akira S. The role of pattern-recognition receptors in innate immunity: update on Toll-like receptors. Nat Immunol. (2010) 11:373–84. doi: 10.1038/ni.1863 20404851

[15] Cai Z Sanchez A Shi Z Zhang T Liu M Zhang D. Activation of Toll-like receptor 5 on breast cancer cells by flagellin suppresses cell proliferation and tumor growth. Cancer Res. (2011) 71:2466–75. doi: 10.1158/0008-5472.CAN-10-1993 PMC3074302 21427357

[16] Varn FS Mullins DW Arias-Pulido H Fiering S Cheng C. Adaptive immunity programmes in breast cancer. Immunology. (2017) 150:25–34. doi: 10.1111/imm.12664 27564847 PMC5341497

[17] Xia B Shan M Wang J Zhong Z Geng J He X. Homeobox A11 hypermethylation indicates unfavorable prognosis in breast cancer. Oncotarget. (2017) 8:9794–805. doi: 10.18632/oncotarget.14216 PMC5354771 28038461

[18] Bedognetti D Ceccarelli M Galluzzi L Lu R Palucka K Samayoa J. Toward a comprehensive view of cancer immune responsiveness: a synopsis from the SITC workshop. J Immunother Cancer. (2019) 7:131. doi: 10.1186/s40425-019-0602-4 31113486 PMC6529999

[19] Batalha S Ferreira S Brito C. The peripheral immune landscape of breast cancer: clinical findings and *in vitro* models for biomarker discovery. Cancers (Basel). (2021) 13:1–28. doi: 10.3390/cancers13061305 PMC8001103 33804027

[20] Huang X Shi Z Wang W Bai J Chen Z Xu J. Identification and characterization of a novel protein ISOC2 that interacts with p16INK4a. Biochem Biophys Res Commun. (2007) 361:287–93. doi: 10.1016/j.bbrc.2007.06.181 17658461

[21] Liu Y Hu Y Xue J Li J Yi J Bu J. Advances in immunotherapy for triple-negative breast cancer. Mol Cancer. (2023) 22:145. doi: 10.1186/s12943-023-01850-7 37660039 PMC10474743

[22] Rizzo A Ricci AD Lanotte L Lombardi L Di Federico A Brandi G. Immune-based combinations for metastatic triple negative breast cancer in clinical trials: current knowledge and therapeutic prospects. Expert Opin Investig Drugs. (2022) 31:557–65. doi: 10.1080/13543784.2022.2009456 34802383

[23] Shumway NM Ibrahim N Ponniah S Peoples GE Murray JL. Therapeutic breast cancer vaccines: a new strategy for early-stage disease. BioDrugs. (2009) 23:277–87. doi: 10.2165/11313490-000000000-00000 19754218

[24] Sayaman RW Saad M Thorsson V Hu D Hendrickx W Roelands J. Germline genetic contribution to the immune landscape of cancer. Immunity. (2021) 54:367–386 e8. doi: 10.1016/j.immuni.2021.01.011 33567262 PMC8414660

[25] Sato Y Goto Y Narita N Hoon DS. Cancer cells expressing toll-like receptors and the tumor microenvironment. Cancer Microenviron. (2009) 2 Suppl 1:205–14. doi: 10.1007/s12307-009-0022-y PMC2756339 19685283

[26] Wang Y Liu S Zhang Y Yang J. Dysregulation of TLR2 serves as a prognostic biomarker in breast cancer and predicts resistance to endocrine therapy in the luminal B subtype. Front Oncol. (2020) 10:547. doi: 10.3389/fonc.2020.00547 32426275 PMC7203473

[27] Scheeren FA Kuo AH van Weele LJ Cai S Glykofridis I Sikandar SS. A cell-intrinsic role for TLR2-MYD88 in intestinal and breast epithelia and oncogenesis. Nat Cell Biol. (2014) 16:1238–48. doi: 10.1038/ncb3058 25362351

[28] Wujcicka W Paradowska E Studzinska M Wilczynski J Nowakowska D. Toll-like receptors genes polymorphisms and the occurrence of HCMV infection among pregnant women. Virol J. (2017) 14:64. doi: 10.1186/s12985-017-0730-8 28340580 PMC5364709

[29] Butkowsky C Aldor N Poynter SJ. Toll−like receptor 3 ligands for breast cancer therapies (Review). Mol Clin Oncol. (2023) 19:60. doi: 10.3892/mco.2023.2656 37424627 PMC10326562

[30] Li D Gu R Yang X Hu C Li Y Gao M. TLR3 correlated with cervical lymph node metastasis in patients with papillary thyroid cancer. Int J Clin Exp Med. (2014) 7:5111–7. PMC4307459 25664012

[31] Yang H Zhou H Feng P Zhou X Wen H Xie X. Reduced expression of Toll-like receptor 4 inhibits human breast cancer cells proliferation and inflammatory cytokines secretion. J Exp Clin Cancer Res. (2010) 29:92. doi: 10.1186/1756-9966-29-92 20618976 PMC2913950

[32] Fang H Ang B Xu X Huang X Wu Y Sun Y. TLR4 is essential for dendritic cell activation and anti-tumor T-cell response enhancement by DAMPs released from chemically stressed cancer cells. Cell Mol Immunol. (2014) 11:150–9. doi: 10.1038/cmi.2013.59 PMC4003380 24362470

[33] Safarzadeh E Mohammadi A Mansoori B Duijf PHG Hashemzadeh S Khaze V. STAT3 silencing and TLR7/8 pathway activation repolarize and suppress myeloid-derived suppressor cells from breast cancer patients. Front Immunol. (2020) 11:613215. doi: 10.3389/fimmu.2020.613215 33679700 PMC7933669

[34] Moreno Ayala MA Gottardo MF Gori MS Nicola Candia AJ Caruso C De Laurentiis A. Dual activation of Toll-like receptors 7 and 9 impairs the efficacy of antitumor vaccines in murine models of metastatic breast cancer. J Cancer Res Clin Oncol. (2017) 143:1713–32. doi: 10.1007/s00432-017-2421-7 PMC11819200 28432455

[35] Urban-Wojciuk Z Khan MM Oyler BL Fahraeus R Marek-Trzonkowska N Nita-Lazar A. The role of TLRs in anti-cancer immunity and tumor rejection. Front Immunol. (2019) 10:2388. doi: 10.3389/fimmu.2019.02388 31695691 PMC6817561

[36] Lu H Yang Y Gad E Inatsuka C Wenner CA Disis ML. TLR2 agonist PSK activates human NK cells and enhances the antitumor effect of HER2-targeted monoclonal antibody therapy. Clin Cancer Res. (2011) 17:6742–53. doi: 10.1158/1078-0432.CCR-11-1142 PMC3206987 21918170

[37] Salaun B Coste I Rissoan MC Lebecque SJ Renno T. TLR3 can directly trigger apoptosis in human cancer cells. J Immunol. (2006) 176:4894–901. doi: 10.4049/jimmunol.176.8.4894 16585585

[38] Fan L Sui XY Jin X Zhang WJ Zhou P Shao ZM. High expression of TLR3 in triple-negative breast cancer predicts better prognosis-data from the Fudan University Shanghai Cancer Center cohort and tissue microarrays. BMC Cancer. (2023) 23:298. doi: 10.1186/s12885-023-10721-9 37005579 PMC10067281

[39] Wen J Zhang J Wu X Yan X Qin X Wang Y. Prognostic and clinicopathological significance of TLR4 expression in patients with breast cancer: a meta-analysis. Front Oncol. (2024) 14:1344130. doi: 10.3389/fonc.2024.1344130 38463226 PMC10920234

[40] Shi Z Cai Z Yu J Zhang T Zhao S Smeds E. Toll-like receptor 11 (TLR11) prevents Salmonella penetration into the murine Peyer patches. J Biol Chem. (2012) 287:43417–23. doi: 10.1074/jbc.M112.411009 PMC3527929 23135279

[41] Shuang C Weiguang Y Zhenkun F Yike H Jiankun Y Jing X. Toll-like receptor 5 gene polymorphism is associated with breast cancer susceptibility. Oncotarget. (2017) 8:88622–9. doi: 10.18632/oncotarget.20242 PMC5687632 29179462

[42] Le Mercier I Poujol D Sanlaville A Sisirak V Gobert M Durand I. Tumor promotion by intratumoral plasmacytoid dendritic cells is reversed by TLR7 ligand treatment. Cancer Res. (2013) 73:4629–40. doi: 10.1158/0008-5472.CAN-12-3058 23722543

[43] Roychowdhury A Jondhale M Saldanha E Ghosh D Kumar Panda C Chandrani P. Landscape of toll-like receptors expression in tumor microenvironment of triple negative breast cancer (TNBC): Distinct roles of TLR4 and TLR8. Gene. (2021) 792:145728. doi: 10.1016/j.gene.2021.145728 34022297

[44] Zhou P Qin J Zhou C Wan G Liu Y Zhang M. Multifunctional nanoparticles based on a polymeric copper chelator for combination treatment of metastatic breast cancer. Biomaterials. (2019) 195:86–99. doi: 10.1016/j.biomaterials.2019.01.007 30623789

[45] Zhang D Zhang G Hayden MS Greenblatt MB Bussey C Flavell RA. A toll-like receptor that prevents infection by uropathogenic bacteria. Science. (2004) 303:1522–6. doi: 10.1126/science.1094351 15001781

[46] Kornilov FD Shabalkina AV Lin C Volynsky PE Kot EF Kayushin AL. The architecture of transmembrane and cytoplasmic juxtamembrane regions of Toll-like receptors. Nat Commun. (2023) 14:1503. doi: 10.1038/s41467-023-37042-6 36932058 PMC10023784

[47] Nagashima H Iwatani S Cruz M Jimenez Abreu JA Uchida T Mahachai V. Toll-like receptor 10 in helicobacter pylori infection. J Infect Dis. (2015) 212:1666–76. doi: 10.1093/infdis/jiv270 PMC4621249 25977263

[48] Xie W Huang Y Xie W Guo A Wu W. Bacteria peptidoglycan promoted breast cancer cell invasiveness and adhesiveness by targeting toll-like receptor 2 in the cancer cells. PloS One. (2010) 5:e10850. doi: 10.1371/journal.pone.0010850 20520770 PMC2877101

[49] Di Lorenzo A Bolli E Ruiu R Ferrauto G Di Gregorio E Avalle L. Toll-like receptor 2 promotes breast cancer progression and resistance to chemotherapy. Oncoimmunology. (2022) 11:2086752. doi: 10.1080/2162402X.2022.2086752 35756841 PMC9225225

[50] Ignacio RMC Gibbs CR Kim S Lee ES Adunyah SE Son DS. Serum amyloid A predisposes inflammatory tumor microenvironment in triple negative breast cancer. Oncotarget. (2019) 10:511–26. doi: 10.18632/oncotarget.26566 PMC6355188 30728901

[51] Green TL Santos MF Ejaeidi AA Craft BS Lewis RE Cruse JM. Toll-like receptor (TLR) expression of immune system cells from metastatic breast cancer patients with circulating tumor cells. Exp Mol Pathol. (2014) 97:44–8. doi: 10.1016/j.yexmp.2014.05.003 24836676

[52] Abdin AA Soliman NA Saied EM. Effect of propranolol on IL-10, visfatin, Hsp70, iNOS, TLR2, and survivin in amelioration of tumor progression and survival in Solid Ehrlich Carcinoma-bearing mice. Pharmacol Rep. (2014) 66:1114–21. doi: 10.1016/j.pharep.2014.07.010 25443743

[53] Mafi S Mansoori B Taeb S Sadeghi H Abbasi R Cho WC. mTOR-mediated regulation of immune responses in cancer and tumor microenvironment. Front Immunol. (2021) 12:774103. doi: 10.3389/fimmu.2021.774103 35250965 PMC8894239

[54] Amirfakhri S Salimi A Fernandez N. Effects of conditioned medium from breast cancer cells on tlr2 expression in nb4 cells. Asian Pac J Cancer Prev. (2015) 16:8445–50. doi: 10.7314/APJCP.2015.16.18.8445 26745099

[55] Khosravi N Hanson ED Farajivafa V Evans WS Lee JT Danson E. Exercise-induced modulation of monocytes in breast cancer survivors. Brain Behav Immun Health. (2021) 14:100216. doi: 10.1016/j.bbih.2021.100216 34589753 PMC8474256

[56] Fan L Zhou P Hong Q Chen AX Liu GY Yu KD. Toll-like receptor 3 acts as a suppressor gene in breast cancer initiation and progression: a two-stage association study and functional investigation. Oncoimmunology. (2019) 8:e1593801. doi: 10.1080/2162402X.2019.1593801 31069157 PMC6492959

[57] Bernardo AR Cosgaya JM Aranda A Jimenez-Lara AM. Synergy between RA and TLR3 promotes type I IFN-dependent apoptosis through upregulation of TRAIL pathway in breast cancer cells. Cell Death Dis. (2013) 4:e479. doi: 10.1038/cddis.2013.5 23370279 PMC3564005

[58] Alshetaiwi H Pervolarakis N McIntyre LL Ma D Nguyen Q Rath JA. Defining the emergence of myeloid-derived suppressor cells in breast cancer using single-cell transcriptomics. Sci Immunol. (2020) 5:1–15. doi: 10.1126/sciimmunol.aay6017 PMC7219211 32086381

[59] Jia D Yang W Li L Liu H Tan Y Ooi S. beta-Catenin and NF-kappaB co-activation triggered by TLR3 stimulation facilitates stem cell-like phenotypes in breast cancer. Cell Death Differ. (2015) 22:298–310. doi: 10.1038/cdd.2014.145 25257174 PMC4291491

[60] Tavora B Mederer T Wessel KJ Ruffing S Sadjadi M Missmahl M. Tumoural activation of TLR3-SLIT2 axis in endothelium drives metastasis. Nature. (2020) 586:299–304. doi: 10.1038/s41586-020-2774-y 32999457 PMC8088828

[61] Huang L Rong Y Tang X Yi K Qi P Hou J. Engineered exosomes as an in *situ* DC-primed vaccine to boost antitumor immunity in breast cancer. Mol Cancer. (2022) 21:45. doi: 10.1186/s12943-022-01515-x 35148751 PMC8831689

[62] Ahmed A Redmond HP Wang JH. Links between Toll-like receptor 4 and breast cancer. Oncoimmunology. (2013) 2:e22945. doi: 10.4161/onci.22945 23526132 PMC3601164

[63] Rajput S Volk-Draper LD Ran S. TLR4 is a novel determinant of the response to paclitaxel in breast cancer. Mol Cancer Ther. (2013) 12:1676–87. doi: 10.1158/1535-7163.MCT-12-1019 PMC3742631 23720768

[64] Yang H Wang B Wang T Xu L He C Wen H. Toll-like receptor 4 prompts human breast cancer cells invasiveness via lipopolysaccharide stimulation and is overexpressed in patients with lymph node metastasis. PloS One. (2014) 9:e109980. doi: 10.1371/journal.pone.0109980 25299052 PMC4192367

[65] Li G Sun Y Huang Y Lian J Wu S Luo D. Fusobacterium nucleatum-derived small extracellular vesicles facilitate tumor growth and metastasis via TLR4 in breast cancer. BMC Cancer. (2023) 23:473. doi: 10.1186/s12885-023-10844-z 37221488 PMC10207721

[66] Afroz R Tanvir EM Tania M Fu J Kamal MA Khan MA. LPS/TLR4 pathways in breast cancer: insights into cell signalling. Curr Med Chem. (2022) 29:2274–89. doi: 10.2174/0929867328666210811145043 34382520

[67] Oblak A Jerala R. Toll-like receptor 4 activation in cancer progression and therapy. Clin Dev Immunol. (2011) 2011:609579. doi: 10.1155/2011/609579 22110526 PMC3216292

[68] Shi M Yao Y Han F Li Y Li Y. MAP1S controls breast cancer cell TLR5 signaling pathway and promotes TLR5 signaling-based tumor suppression. PloS One. (2014) 9:e86839. doi: 10.1371/journal.pone.0086839 24466264 PMC3900661

[69] Shi M Zhang Y Liu L Zhang T Han F Cleveland J. MAP1S protein regulates the phagocytosis of bacteria and toll-like receptor (TLR) signaling. J Biol Chem. (2016) 291:1243–50. doi: 10.1074/jbc.M115.687376 PMC4714212 26565030

[70] Gonzalez C Williamson S Gammon ST Glazer S Rhee JH Piwnica-Worms D. TLR5 agonists enhance anti-tumor immunity and overcome resistance to immune checkpoint therapy. Commun Biol. (2023) 6:31. doi: 10.1038/s42003-022-04403-8 36635337 PMC9837180

[71] Shi S Xu C Fang X Zhang Y Li H Wen W. Expression profile of Toll−like receptors in human breast cancer. Mol Med Rep. (2020) 21:786–94. doi: 10.3892/mmr.2019.10853 PMC6947885 31789409

[72] Yin T He S Wang Y. Toll-like receptor 7/8 agonist, R848, exhibits antitumoral effects in a breast cancer model. Mol Med Rep. (2015) 12:3515–20. doi: 10.3892/mmr.2015.3885 26043701

[73] Hosoya T Sato-Kaneko F Ahmadi A Yao S Lao F Kitaura K. Induction of oligoclonal CD8 T cell responses against pulmonary metastatic cancer by a phospholipid-conjugated TLR7 agonist. Proc Natl Acad Sci U.S.A. (2018) 115:E6836–44. doi: 10.1073/pnas.1803281115 PMC6055176 29967183

[74] Huang S Wen T Wang J Wei H Xiao Z Li B. Nanoparticle-integrated dissolving microneedles for the co-delivery of R848/aPD-1 to synergistically reverse the immunosuppressive microenvironment of triple-negative breast cancer. Acta Biomater. (2024) 176:344–55. doi: 10.1016/j.actbio.2024.01.009 38244662

[75] Ye J Ma C Hsueh EC Dou J Mo W Liu S. TLR8 signaling enhances tumor immunity by preventing tumor-induced T-cell senescence. EMBO Mol Med. (2014) 6:1294–311. doi: 10.15252/emmm.201403918 PMC4287933 25231413

[76] Merrell MA Ilvesaro JM Lehtonen N Sorsa T Gehrs B Rosenthal E. Toll-like receptor 9 agonists promote cellular invasion by increasing matrix metalloproteinase activity. Mol Cancer Res. (2006) 4:437–47. doi: 10.1158/1541-7786.MCR-06-0007 16849519

[77] Ilvesaro JM Merrell MA Li L Wakchoure S Graves D Brooks S. Toll-like receptor 9 mediates CpG oligonucleotide-induced cellular invasion. Mol Cancer Res. (2008) 6:1534–43. doi: 10.1158/1541-7786.MCR-07-2005 18922969

[78] Berger R Fiegl H Goebel G Obexer P Ausserlechner M Doppler W. Toll-like receptor 9 expression in breast and ovarian cancer is associated with poorly differentiated tumors. Cancer Sci. (2010) 101:1059–66. doi: 10.1111/j.1349-7006.2010.01491.x PMC3188854 20156214

[79] Qiu J Shao S Yang G Shen Z Zhang Y. Association of Toll like receptor 9 expression with lymph node metastasis in human breast cancer. Neoplasma. (2011) 58:251–5. doi: 10.4149/neo_2011_03_251 21391743

[80] Tuomela J Sandholm J Karihtala P Ilvesaro J Vuopala KS Kauppila JH. Low TLR9 expression defines an aggressive subtype of triple-negative breast cancer. Breast Cancer Res Treat. (2012) 135:481–93. doi: 10.1007/s10549-012-2181-7 22847512

[81] Gonzalez-Reyes S Marin L Gonzalez L Gonzalez LO del Casar JM Lamelas ML. Study of TLR3, TLR4 and TLR9 in breast carcinomas and their association with metastasis. BMC Cancer. (2010) 10:665. doi: 10.1186/1471-2407-10-665 21129170 PMC3009680

[82] Wu J Li S Yang Y Zhu S Zhang M Qiao Y. TLR-activated plasmacytoid dendritic cells inhibit breast cancer cell growth *in vitro* and *in vivo* . Oncotarget. (2017) 8:11708–18. doi: 10.18632/oncotarget.14315 PMC5355297 28052019

[83] Wei X Liu L Li X Wang Y Guo X Zhao J. Selectively targeting tumor-associated macrophages and tumor cells with polymeric micelles for enhanced cancer chemo-immunotherapy. J Control Release. (2019) 313:42–53. doi: 10.1016/j.jconrel.2019.09.021 31629039

[84] Gatti-Mays ME Balko JM Gameiro SR Bear HD Prabhakaran S Fukui J. If we build it they will come: targeting the immune response to breast cancer. NPJ Breast Cancer. (2019) 5:37. doi: 10.1038/s41523-019-0133-7 31700993 PMC6820540

[85] Akhand SS Liu Z Purdy SC Abdullah A Lin H Cresswell GM. Pharmacologic inhibition of FGFR modulates the metastatic immune microenvironment and promotes response to immune checkpoint blockade. Cancer Immunol Res. (2020) 8:1542–53. doi: 10.1158/2326-6066.CIR-20-0235 PMC7710538 33093218

[86] Zhang C Ben A Reville J Calabrese V Villa NN Bandyopadhyay M. Immunotherapeutic impact of toll-like receptor agonists in breast cancer. Anticancer Agents Med Chem. (2015) 15:1134–40. doi: 10.2174/1871520615666150518092547 25980815

[87] Braunstein MJ Kucharczyk J Adams S. Targeting toll-like receptors for cancer therapy. Target Oncol. (2018) 13:583–98. doi: 10.1007/s11523-018-0589-7 30229471

[88] Zhu Y Zhu X Tang C Guan X Zhang W. Progress and challenges of immunotherapy in triple-negative breast cancer. Biochim Biophys Acta Rev Cancer. (2021) 1876:188593. doi: 10.1016/j.bbcan.2021.188593 34280474

[89] Li Y Zhang H Merkher Y Chen L Liu N Leonov S. Recent advances in therapeutic strategies for triple-negative breast cancer. J Hematol Oncol. (2022) 15:121. doi: 10.1186/s13045-022-01341-0 36038913 PMC9422136

